# Molecular and Cellular Mechanisms of Corneal Scarring and Advances in Therapy

**DOI:** 10.3390/ijms24097777

**Published:** 2023-04-24

**Authors:** Matthias Fuest, Vishal Jhanji, Gary Hin-Fai Yam

**Affiliations:** 1 Department of Ophthalmology, RWTH Aachen University, 52074 Aachen, Germany; 2 Cornea Bank Aachen, RWTH Aachen University, 52074 Aachen, Germany; 3 Department of Ophthalmology, University of Pittsburgh, Pittsburgh, PA 15213, USA; jhanjiv@pitt.edu; 4 Singapore Eye Research Institute, Singapore 169856, Singapore; 5 McGowan Institute for Regenerative Medicine, Pittsburgh, PA 15213, USA

On the basis of WHO global blindness data, it may be stated that 23 million people globally suffer from unilateral corneal blindness, while 4.9 million are bilaterally blind [1]. Corneal transplantation with donor allograft (i.e., keratoplasty) has been the mainstay of corneal blindness and scarring treatment for a century. Today, the volume of corneal transplantations (either in the form of penetrating or lamellar keratoplasty) is continuously growing. Each year, about 30,000 corneal transplants are performed in the European Union, and about 46,000 in the USA (Eye Bank Association of America, European Corneal Transplant Register, 2021). However, a global shortage of donor corneas still leaves some 12.7 million patients on the waiting lists, and only 1 in 70 patients can gain access to a transplantable donor cornea [2]. Penetrating keratoplasty (PK) is very efficient in recovering patient vision. However, the rejection rate can be up to 15% in inflamed eyes, leading to about an approximately 10% rate of failure within two years [3]. In the longer term, the allograft’s viability drops and the usual failure rate is about 50% grafts for the 10 to 15 years after surgery. In the case of patients with immunological disorders, such as Stevens–Johnson syndrome, severe corneal vascularization or damage by alkali burn, the allograft toleration level drops further. The corneal tissue function cannot be recovered, and the success rate is low, especially when a second graft is required [4]. As the donor cornea shortage is a crucial global issue, many research groups are investigating strategies to regulate corneal wound healing and control scar development in order to decrease the need for corneal transplants in the future.

Scar formation during corneal wound healing is a significant clinical problem. It is the fourth leading cause of blindness globally according to the World Health Organization (https://www.who.int/news-room/fact-sheets/detail/blindness-and-visual-impairment; assessed on 30 March 2023), accounting for approximately ~4% of all cases of avoidable vision loss. Clinically, attempts to control corneal scarring have mostly involved the use of steroids or mitomycin C (MMC) [5]. While effective at decreasing myofibroblast differentiation and haze, these two compounds exhibit significant side-effects. In the case of MMC, toxicity and DNA damage to stromal keratocytes and corneal endothelial cells (CEndoC) can bear long-term negative consequences for ocular health [6]. In recent years, various efforts have been undertaken to investigate alternative medical and pharmacological strategies to impede corneal scarring [7,8,9,10]; however, the success has been limited. Hence, it is important to increase the level of scientific knowledge about the pathophysiological mechanisms of corneal wound healing in order to find ways to avoid scar formation. The immune-privileged nature of the cornea has attracted the attention of many researchers, serving as a possible motivation for the development of new therapies. There has been increase in research using cell-based and cell-free strategies, bioengineered constructs, and scaffolds, as well as corneal protheses, to impede corneal scarring and to retrieve vision from scarred corneas [11,12,13,14].

This Special Issue of the *International Journal of Molecular Sciences*, entitled “Molecular and Cellular Mechanisms of Corneal Fibrosis/Scarring and Advances in Therapy”, provides an overview of the molecular and cellular mechanisms associated with corneal fibrosis and scarring. Additionally, this publication surveys the latest research into the treatment modalities of corneal scarring and their short- and long-term consequences. It includes a total of 9 contributions: 7 original articles and 2 reviews.

1. Talpan et al., presented an interesting report on the use of human platelet lysate (HPL) as a xeno-free substitute for fetal bovine serum (FBS) in human donor cornea organ cultures [15]. Xenogenic FBS is habitually used during the storage and transport of donor corneal tissue, as well as during the preparation of cell-based and bioengineered corneal therapy approaches. However, FBS has been associated with anaphylactic, inflammatory, and pro-fibrogenic reactions, and international authorities encourage the use of xeno-free alternatives. The study showed that CEndoC loss was significantly lower in 2% HPL than in 2% FBS. Interestingly, the next generation sequencing revealed the upregulation of cytoprotective, anti-inflammatory and anti-fibrotic genes and the downregulation of pro-inflammatory/apoptotic genes in the HPL-cultured corneas. Hence, the HPL medium could prove favorable for use in cornea organ culture, transplantation, cell- and tissue-engineered therapies.
2. In the second article by Talpan et al., the authors evaluated the small molecules (AFM)—caffeine, curcumin and pirfenidone—to study their inhibitory effects on the transformation of human stromal keratocytes into scar-inducing myofibroblasts [16]. Using donor keratocytes, the authors optimized the chemicals’ dosage effect, without cytotoxicity, in order to significantly reduce myofibroblasts (αSMA expression) under TGFβ1 induction. These results are promising in terms of the efforts to suppress keratocyte–myofibroblast conversion during scar tissue formation. However, further studies will be necessary to investigate the ideal application in tissue engineering and translational purposes.
3. Disulfiram (DSF), a drug treatment for alcoholism, has been found to have FROUNT (a macrophage stimulator) inhibitory activity. Ikebukuro et al. showed that DSF eye drops reduced macrophage accumulation in a rat corneal alkali burn model [17]. After suffering alkali burns, treatment of 0.5% DSF eye drops, performed twice daily significantly, inhibited macrophage infiltration. However, it made no difference in neutrophils. The expression of macrophage-associated cytokines was decreased, thereby suppressing corneal scarring and neovascularization. These findings make DSF an interesting candidate for further investigation and clinical trials.
4. Limbus-derived stromal/mesenchymal stem cells (LMSC) are vital for corneal stromal homeostasis and wound healing. Despite multiple pre-clinical and clinical studies reporting the potency of LMSC in suppressing corneal inflammation and scarring, the molecular basis for the ability of LMSC remains unknown. Tavakkoli et al. characterized the cultured LMSC at the stages of initiation (LMSC-P0) and pure population (LMSC-P3) [18]. Their RNA-seq study has identified differentially expressed genes (DEGs) in comparison to the native limbus, cornea, and scleral tissue. Among 28,000 genes detected, 7800 DEGs were involved in Wnt and TGFβ signaling pathways, as well as 16 other biological processes, including apoptosis, cell motility, tissue remodeling, and stem cell maintenance. Two hundred and fifty-four genes were related to wound healing pathways. COL5A1 and TIMP1 were exclusively upregulated in LMSC-P. Hence, these findings provide new insights and highlight the importance of Wnt and TGFβ signaling as well as specific genes for LMSC-mediated tissue healing.
5. Various preclinical studies have shown that cell-based treatments, performed using either human corneal stromal stem cells (CSSC) or stromal keratocytes, suppress corneal scarring at lower levels. Jhanji et al. presented an interesting study using a combined cell-based treatment in a mouse model of anterior stromal injury [19]. Their results demonstrated that the effect of CSSC for suppressing corneal opacities was augmented by an additional intrastromal keratocyte injection, resulting in better corneal clarity. These in vivo effects were substantiated by further downregulation of the expression of fibrosis genes and the restoration of stromal fibrillar organization and regularity. The promising results confirmed the authors’ hypothesis that the immuno-regulatory nature of CSSC was effective at controlling tissue inflammation and delaying the onset of fibrosis. Additionally, the researchers performed a subsequent intrastromal administration of keratocyte-deposited collagens and stromal-specific proteoglycans to recover a native stromal matrix. Hence, a combined stromal stem cell and keratocyte treatment could achieve a higher clinical efficacy and restore corneal transparency, when compared to the use of a single-cell type of therapy.
6. The latest research affects not only the stroma, but also the ocular surface and the corneal endothelium. Sasamoto et al. revealed a novel function of ten-eleven translocation dioxygenase 2 (TET2) via 5-hydroxymethylcytosine (5hmC) in the epigenetic regulation of corneal epithelial gene expression [20]. Both 5hmC and its generating enzyme TET2 were highly expressed in terminally differentiated epithelial cells. The TET2 knockdown in epithelial cells transcriptionally repressed epithelial differentiation. Novel TET2-controlled genes were identified by RNA-seq and genome-wide reduced representation hydroxymethylation profiling analyses. These results thus reveal a novel role of TET2 in the epigenetic regulation of corneal epithelial differentiation and suggest further research on TET2-inducing strategy to treat abnormal epithelial maturation associated with corneal diseases.
7. Cell injection therapy is an emerging alternative with which to treat corneal endothelial dysfunction, e.g., in bullous keratopathy. However, establishing a standard culture procedure that provides appropriate cell yield while retaining the functional features remains a challenge. The article by Bandeira et al. demonstrated that human CEndoC isolation with collagenase rendered better cell morphology after adhesion to culture surface when compared to EDTA [21]. ROCK inhibitor Y-27632 supplementation resulted in a 2.6-fold increase in the final cell yield. Cell delivery was successful with two systems (superparamagnetic embedding and cell suspension) and the treatment reduced corneal edema and opacity in an ex vivo human cornea culture and in vivo rabbit model of endothelial keratopathy. Though the results are very promising, additional work, such as CEndoC quality and functional property as well as cell manufacturing under GMP conditions, is required before this treatment can advance to application in cell therapy in humans.
8. Ultrathin stromal laminar tissues obtained from lenticule-based refractive correction procedures, such as small incision lenticule extraction (SMILE), are accessible and novel sources of native collagen-rich scaffolds with high mechanical strength, biocompatibility, and transparency. After customization (including decellularization), the SMILE lenticules constitute acellular scaffold niche to repopulate cells, including stromal keratocytes and stem cells, with functional phenotypes. The intrastromal transplantation of the engineered cell/tissue composite regenerated native-like corneal stromal tissue and restored corneal transparency. The review by Santra et al. highlighted the current status of ECM scaffold-based corneal engineering with cells, along with the development of drug and growth factor delivery and elucidated the potential uses of stromal lenticule scaffolds in regenerative therapeutics [22].
9. Accumulating evidence in different cell models has highlighted the role of extracellular vesicles (EVs) and exosomes in modulating cell signaling through paracrine mechanisms and their therapeutic potential. In recent years, understanding the pathological and therapeutic EV mechanisms of action in the context of corneal biology has been a topic of increasing interest. In the review of Yeung et al., the authors discussed the clinical relevance of corneal fibrosis and how corneal stromal cells contributed to wound repair and corneal scarring. Furthermore, they elaborated on EV characterization and the pathological and therapeutic roles of EVs in corneal scarring [23].

In summary, this Special Issue gives an overview of molecular and cellular strategies and provides new insights into fibrosis and scar tissue formation in corneas. It also discusses the latest research on novel treatment modalities, focusing on the application of modulating substances, EVs and corneal cell types as well as scaffold-based regenerative therapeutics. These studies provide strong evidence about the future corneal regeneration which is already started. Contemporary medicine is awash with the emerging techniques that target the replacement of human corneal tissues and improvement of treatment outcomes. Hopefully these novel strategies will be able reduce the immunological issues, albeit in a way that provides with greater control, improved functional recovery, and a lower rate of induction of comorbidities and adverse effects.

## Data Availability

Data are available on request.
